# Can what have we learnt about BCG vaccination in the last 20 years help us to design a better tuberculosis vaccine?

**DOI:** 10.1016/j.vaccine.2021.01.068

**Published:** 2022-03-08

**Authors:** Hazel M. Dockrell, Egle Butkeviciute

**Affiliations:** Department of Infection Biology, Faculty of Infectious and Tropical Diseases, London School of Hygiene & Tropical Medicine, Keppel Street, London WC1E 7HT, UK

**Keywords:** BCG, BCG vaccination, Tuberculosis, TB vaccines, Trained immunity

## Abstract

The BCG vaccine will, in 2021, have been in use for 100 years. Much remains to be understood, including the reasons for its variable efficacy against pulmonary tuberculosis in adults. This review will discuss what has been learnt about the BCG vaccine in the last two decades, and whether this new information can be exploited to improve its efficacy, by enhancing its ability to induce either antigen-specific and/or non-specific effects. Many factors affect both the immunogenicity of BCG and its protective efficacy, highlighting the challenges of working with a live vaccine in man, but new insights may enable us to exploit better what BCG can do.

## Highlights

• The BCG vaccine provides variable protection against tuberculosis.
• Correlates of protection remain elusive, but IFNγ can measure immunogenicity.
• BCG vaccination induces innate immune training as well as antigen-specific immunity.
• Many factors may contribute to the variable responses to BCG vaccination.
• Prior BCG vaccination or factors modulating its efficacy may affect new TB vaccines.
• Innate training may also provide non-specific protection against infectious diseases.
• New TB vaccines should not lose BCG's beneficial non-specific effects.

## 1 The BCG vaccine is capable of inducing protection against tuberculosis in some groups and settings

It is often stated that the BCG vaccine does not provide protection against tuberculosis. This is not true. The systematic review published by Mangtani et al. [1] included randomised controlled trials investigating whether BCG vaccination induced protection against tuberculosis. BCG vaccination is protective in some age groups and in some settings – in neonates against pulmonary and disseminated forms of tuberculosis, and at latitudes of 40° and above it gives better protection against pulmonary disease than in vaccinees living closer to the equator. In school age children protection was stronger if vaccination was restricted to those who were skin test negative to PPD using the Mantoux skin test [1]. These observations support the generally held view that exposure to other mycobacteria can reduce the protection induced with BCG vaccination through either masking the protection that BCG induces, or by blocking multiplication of the live BCG thereby preventing it from inducing protection [2], a consensus that has strengthened in the last 20 years. Overall, it is not correct to say that the BCG vaccine is unable to protect – as it can protect infants and young children against disseminated forms of TB, and adults against pulmonary TB in some circumstances [1]. Most of the world’s children receive BCG vaccination with two-thirds of those countries giving BCG vaccination estimated to have >90% vaccine coverage [3]. BCG vaccination is recommended by the WHO to be given shortly after birth, however, when vaccine coverage is usually assessed at 1 year of age, in some settings many infants have been vaccinated later than the WHO recommends [4], [5]. It is also clear that this wide vaccine coverage has been insufficient to control the spread of tuberculosis. Given that in 2019, there were 10 million individuals diagnosed with tuberculosis (TB) and 1.4 million deaths [6], we need an improved TB vaccine or vaccination regimen [7].

## 2 BCG-induced protective immunity can be long-lived – but this may depend on the type of immunity being measured

Another comment often made about the BCG vaccine is that it fails to induce long-lasting immunity, generally assumed to be mediated by classical T-cell memory. Again, this is not correct, as despite concerns about the induction of long-term immunological memory by BCG in mice, in certain settings, BCG can induce very long-lived protection in man – for as long as 50–60 years in Alaskan natives and American Indians [8]. Although many studies have not included longer term follow-up, the metanalysis by Abubakar et al. [9] identified one trial and four observational studies where protection lasted for 15 years or more. A recent retrospective population-based cohort analysis of BCG vaccination studies in Norway also found 58% protection against pulmonary tuberculosis 10–19 years after vaccination; however, this effect was diminished at later time-points [10].

Such longevity might be possible for antigen-specific immune responses as a result of antigen-specific T-cell memory but so far there is no evidence that non-specific protection as discussed below can last so long. The effects of non-specific trained immunity have so far only been shown to last for several months and to wane by 12 months after BCG vaccination [11], although longer effects might result from the epigenetic reprogramming of cells in the bone marrow [12]. Observational studies have suggested that longer-term non-specific effects can persist in individuals for more than a few years after vaccination; one Danish case-cohort study found that BCG vaccination was associated with protection against natural deaths (but not against accidental deaths, murders or suicides) for decades [13].

## 3 BCG vaccines are variable in composition

There is evidence that different strains of the BCG vaccine can induce varying degrees of T-cell immunity to mycobacterial or heterologous antigens. Infant BCG vaccination studies in Uganda, Nigeria, South Africa and Australia suggested that BCG Denmark may induce higher proportions than BCG Bulgaria or BCG Russia of single or multiple cytokine producing CD4+ T-cells responding to PPD, BCG or heterologous antigens and higher cytokine production by these cells [14], [15], [16]. In Australia, vaccination with BCG Japan outperformed both BCG Russia and BCG Denmark in terms of Th1 cytokine, IL-10, MCP-1 or MIP-1β production in response to mycobacterial antigens [16]. In Brazil, not only was the extent of cytokine production by healthy adult peripheral blood and umbilical cord mononuclear cells different in response to BCG Moreau, BCG Denmark and BCG Pasteur but also the rates of apoptosis: BCG Moreau induced the strongest cytokine production and the greatest degree of apoptosis [17]. Collectively, these studies suggest that different strains of BCG can induce differing classical immune responses.

There are some subtleties here though: any potential strain-specific antimycobacterial or heterologous effects of BCG might be susceptible to confounding, such as delivery route (discussed below) or the number of viable bacilli in the vaccine. *Mycobacterium bovis* BCG can be tricky to grow, even for experienced vaccine producers, and the proportion of live and dead bacilli can vary in different vaccine batches. This makes it hard to compare different BCG strains directly. Even if grown and prepared in exactly the same way, which not all BCG vaccines are, the rate of growth can also vary. A study by Biering-Sørensen et al. [18] showed that slower growing batches resulted in greater vaccination site scarring and increased cytokine production in response to mycobacterial or heterologous stimuli. In another study, different strains of BCG were found to differ in proportions of viable bacilli and to induce divergent cytokine profiles in whole blood from newborns and adults [19]. Interestingly, the number of viable BCG bacilli in this study correlated with levels of GM-CSF, PDGF-AB/BB, IL-1β, TNFα and IFNγ, cytokines known to have roles in antimycobacterial and trained immunity [19], suggesting that the viability of BCG might affect the degree of innate training. Indeed, gamma-irradiation of BCG decreased its ability to induce trained immunity and related cytokine production *in vitro,* although it did not abolish training completely [20].

Potential influences of BCG strains on heterologous downstream effects may be even more difficult to capture. While in normal birth weight infants from Guinea-Bissau BCG Denmark was associated with higher rates of scar formation compared to BCG Russia, there was no significant difference in rates of health consultations between infant groups vaccinated with these two BCG strains [21]. In another study in Guinea-Bissau, no significant differences in morbidity or mortality by 6 weeks of age were observed in newborns given BCG Russia, BCG Denmark and BCG Japan [22]. This suggests that if different strains of BCG affect the non-specific effects induced by BCG, the impact is likely to be limited, although more studies are needed.

In summary, whatever BCG does, live BCG usually does it better than dead bacilli – and this includes not only protection but induction of non-specific trained immunity. Despite these differences in immunogenicity and in composition, the different strains of BCG were not found to be associated with protection against TB in the Mangtani systematic review [1].

## 4 What has been learnt about the immunogenicity of BCG vaccination?

If the BCG vaccine is given to either adolescents or infants in the UK, strong T-cell responses to cross-reactive mycobacterial antigens such as PPD are induced. The last two decades have largely been the era of cytokines for measurement of immunogenicity, with a focus on the measurement of IFNγ secretion.

Comparisons of different geographic settings in a series of trials in adolescents and young adults showed that whereas BCG vaccination induced protection against pulmonary tuberculosis in the UK, in Malawi it failed to induce any protection against tuberculosis (although it did induce some protection against leprosy) [23]. PPD stimulation of diluted whole blood samples from UK adolescents showed minimal IFNγ production prior to BCG vaccination, and a marked increase that was greatest at 3 but that remained strong at 12 months following vaccination [24]. In contrast, in Malawi most adolescents and young adults were pre-sensitised to PPD before BCG vaccination and did not show significant increases in response following BCG vaccination. The ability to make a strong IFNγ response in such assays is associated with changes in DNA methylation [25]. When South African infants progressing to a diagnosis of TB were stratified into groups of high, medium or low IFNγ responders using an ELISPOT assay in which PBMC were stimulated with BCG, the high responders showed the slowest rate of progression to TB [26]. BCG vaccination of UK infants can induce polyfunctional T-cells making IFNγ, TNFα and IL-2 [27], a cell type also attracting much interest as a possible correlate of protection, but in a cohort of South African infants there was no association of these responses with progression to TB disease [28]. T-cell responses are needed though, as shown by how susceptible those with HIV infection are to *M. tuberculosis* infection, or the rapidly progressive infections seen in SCID mice, as well as the increased mycobacterial growth in mice lacking the ability to produce or respond to IFNγ; in mice and in man there are similar examples of genetic mutations in the IFNγ-IL-12 axis resulting in susceptibility to mycobacterial disease [29]. IFNγ provides valuable information about immunogenicity and may play a role in protection but measuring it alone has not delivered a confirmed correlate of protection. A number of other immunological components, such as various cell types, antibodies and cytokines have been proposed to be associated with protection against tuberculosis (Table 1), but confirmed correlates of protection are still needed.

**Table 1:** Immunological components associated with protection against TB.

**Immunological component**	**Association with protection against TB**	**Study**	**References**
***Cellular components***			
**BCG-responsive high-IFNγ producing PBMCs**	Lower risk of progression to TB disease	BCG-vaccinated infants	[26]
**CD4+IFNγ+TNFα+IL-2+T-cells** **Th17 cells**	Enhanced inhibition of BCG growth in MGIT	BCG-vaccinated infants	[27]
**CD4+ central memory T-cells**	Enhanced inhibition of BCG growth in MGIT	*M. tuberculosis* exposed uninfected individuals	[34]
**CD4+IFNγ+TNFα+ T-cells**	Control of *M. tuberculosis* induced lung pathology at study week 6	BCG-vaccinated NHPs	[53]
**CD4+ T-cells:** **CD154+IFNγ+IL-2+TNFα+** **CD154+IL-2+TNFα+** **CD8+ T-cells:** **IFNγ+TNFα+IL-2+** **IFNγ+TNFα+** **Peak CD4+ and CD8+ T-cell counts**	Reduction of thoracic *M. tuberculosis* burden	BCG-vaccinated NHPs	[54]
**CD4+PD-1+KLRG1- T-cells**	Reduction of *M. tuberculosis* burden in lungs and spleen	BCG-vaccinated mice	[57]
**Epigenetically reprogrammed monocytes**	Reduction of *M. tuberculosis* burden in lungs, spleen and bone marrow	BCG-vaccinated and non-vaccinated murine parabiont and adoptive bone marrow transplant models	[12]
**NK cells**	Enhanced inhibition of BCG growth in MGIT	Historically BCG-vaccinated adults	[30], [31]
**B-cells, CXCL10+ CD14_dim_ monocytes**	Enhanced inhibition of BCG growth in MGIT	*M. tuberculosis* exposed uninfected individuals	[34]
**Neutrophils**	Reduction of *M. tuberculosis* burden in the lungs	BCG-vaccinated mice	[83]
***Soluble components***			
**IFNγ**	Control of mycobacterial infection	Human and mice gene deficiencies	[29]
**CXCL9, CXCL10**	Enhanced inhibition of BCG growth in MGIT	*M. tuberculosis* exposed uninfected individuals	[34]
**IL-1β, TNFα, IL-6**	Elevated		
**IL-10** **αPPD-IgA**	Reduction of pulmonary and extrathoracic *M. tuberculosis* burden	BCG-vaccinated NHPs	[59]
**αAg85A-IgG**	Lower risk of progression to TB disease	BCG-vaccinated infants	[26]
**αAM-IgG, αAM-, αLAM-IgM**	Enhanced survival of mice infected with *M. tuberculosis*	*M. tuberculosis* infection in mice	[103]
	Improved clearance of LAM from the circulation and spleen	Exogenous LAM challenge in mice	
**α19-kDa-IgG**	Negative correlation between DTH responses to PPD and α19-kDa-IgG levels	Factory workers unexposed to TB	[104]
**αAM-, αLAM-, αHBHA-, α16-kDa-α-crystalin-, and αMPB83-IgG, anti-mycobacterial IgG, IgA**	Reduced susceptibility to TB or progression to disease	Murine or NHP *M. tuberculosis* infection, passive serum or polyclonal IgG transfer, B-cell deficiency models, functional assays	[105]

**Abbreviations:**

AM – arabinomannan

DTH – delayed-type hypersensitivity

HBHA – heparin binding hemagglutinin

MPB83 – mycobacterial cell surface lipoprotein

LAM – lipoarabinomannan

NHP – non-human primate

**NB:***The list of studies or reviews presented in this table is not comprehensive*.

## 5 Can measuring mycobacterial growth inhibition directly provide a better estimate of protection?

Mycobacterial growth inhibition (MGI) assays have recently been exploited to investigate the association between immunity and the ability to restrict mycobacterial growth following BCG vaccination. They can also provide a system in which the contributions of various cells and cytokines can be dissected.

UK infants showed a marked induction of MGI following BCG vaccination [27]. In healthy adults, historical BCG vaccination was associated with improved mycobacterial growth inhibition *ex vivo* on its own or in the presence of isoniazid or rifampicin [30]. Interestingly, this study detected a possible association between NK cell frequency and inhibition of mycobacterial growth, with a tendency for higher proportions of NK cells in BCG-vaccinated individuals. Further analyses showed that while overall and cytotoxic NK cell frequencies were associated with *ex vivo* inhibition of mycobacterial growth in BCG-naïve individuals, cytokine-producing NK cell responses correlated with control of mycobacterial growth in BCG-vaccinated individuals [31]. BCG vaccination has been shown to enhance NK cell activation and cytokine production in response to mycobacterial or heterologous stimuli in infants and adults, an effect that lasted 3 to 4 months [32], [33]. BCG also protected SCID mice from lethal *Candida* infection with a partial role demonstrated for NK cells [33].

Activated monocytes or macrophages are considered protective against TB and their efficiency in containing mycobacterial infections has also been explored in mycobacterial growth inhibition assays. A study by Joosten et al. found that enhanced secretion of CXCL9 and CXCL10 by non-classical monocytes was associated with greater mycobacterial growth inhibition in individuals who were exposed to TB but not infected, compared to TB patients or healthy controls, although central memory T-cell responses and B-cell frequencies were also associated with control of mycobacterial growth [34]. PBMCs from those exposed to TB also showed some features consistent with innate immune training, e.g. elevation of innate immune cytokines IL-1β, TNFα or IL-6 in response to BCG stimulation and higher CXCL10 production in response to heterologous stimuli, although monocyte TNFα was not associated with improved mycobacterial growth inhibition.

## 6 Other explanations for variable responses to BCG

The complexity of measuring such vaccine-induced immune responses in the “real world” is very considerable. Immune status is affected by our environment, health, nutrition, microbiome, age, and more [35]. Marked differences in the IFNγ and broader cytokine responses following *in vitro* stimulation of diluted whole blood with PPD were observed between Malawian and UK infants who were BCG vaccinated 3 or 12 months previously [36], [37]. A study of cytokine responses in diluted whole blood cultures stimulated with PPD for 6 days in Ugandan infants given BCG at birth showed the development of immune responses that peaked 4–10 weeks post vaccination, but with considerable individual variation, and some infants failed to make a detectable cytokine response [38]. The literature on how delaying BCG vaccination affects the immunogenicity of BCG vaccination has not shown any consistent improvement with delayed vaccination [39]. The genetics of the vaccinees, other vaccines they are given, nutrition, seasonality and more, will influence these responses. However, certain additional factors have received more attention in the last decade.

## 7 Maternal influences on the response to vaccination in their infants

Newborn infants receiving BCG vaccination should be immunologically naïve, and any confounding effects of environmental or non-tuberculous mycobacteria should not be present. However, young infants may have an immature immune system that has been influenced by their mothers’ immune or infection status [40], [41]. For example, latent TB infection (LTBI) in a woman might influence how her baby responds to BCG vaccination. In Uganda, cytokine responses in BCG-vaccinated infants showed no association with the mothers’ LTBI status [38]. This is perhaps surprising, as mycobacterial antigens might have crossed the placenta and induced either sensitisation or tolerance in the infant. Other common infections, such as malaria or other parasitic infections in the mothers during pregnancy can have broad immunomodulatory effects on the immune system of the newborns/infants. Although helminth infections in the mother had limited effects on the response to BCG in Uganda [42], viral infections such as CMV alter CD8 T-cells and rate of progression to TB in infants [26], [43].

The BCG vaccination status of the mother may also have some effects on Th2 cytokine responses in BCG vaccinated infants to *M. tuberculosis* culture filtrate proteins or cord blood IL-10, IFNγ or immune cell growth factor responses to innate stimuli [42], [44]. A possible beneficial association of previous maternal immunisation with BCG and lower rates of parent-reported infections was found in infants at 0–3 months of age in the Danish BCG study [45], and maternal BCG scar was also associated with lower infant mortality risk in Guinea-Bissau [46]. Whether this reflects an as yet unknown biological mechanism or was associated with confounding healthcare practices within a family is not clear.

## 8 Would BCG be more protective if given by another route?

Although when first used in 1921 the BCG vaccine was given orally, it is now given intradermally. One area of recent and active research is whether BCG (and other novel TB vaccines) might be more protective if given by routes other than the standard intradermal route. Intradermal vaccination is tricky and well-trained staff are needed to give intradermal vaccines such as BCG. Some countries have therefore used a multipuncture device to deliver BCG; this has the added benefit of reducing scarring which in some cultures is regarded by parents/guardians as of major importance. For example, in Japan and South Korea BCG Japan has been delivered percutaneously with a multi-puncture device, but skin test responses and IFNγ responses to PPD in Korean children aged 4–7 years given BCG Pasteur intradermally or BCG Tokyo by multipuncture device were comparable [47]. A larger trial of BCG given percutaneously or intradermally in South Africa found that there was no difference in the protective efficacy of BCG given by these routes [48], [49]. A strength of this South African study was that the same BCG strain (Japan 172) was given by both routes. Presence of a BCG scar is often used as a proxy for BCG vaccination history, although scars can disappear over time and not all of those vaccinated develop a scar. Both the presence and the size of BCG scar in children that have received BCG vaccination has been associated with improved survival indicating non-specific protection [50], [51].

Studies in animal models have shown that BCG can be more protective if given intravenously rather than by other routes. Early studies in which BCG was given to non-human primates (NHPs) were published as long ago as 1970 [52] but there has been a recent revival of interest in giving BCG by this route. Vaccinating Rhesus macaques by the intravenous route induced better protection than giving BCG intradermally, or intradermally with boosting by intratracheal administration [53]. A larger study in Rhesus macaques used positron emission tomography–computed tomography (PET–CT) imaging with ^18^F-fluorodeoxyglucose, confirming the improved protection given by intravenous BCG, and showed that 9/10 animals given BCG intravenously failed to show any lung lesions [54]. In another study, giving BCG to mice intravenously was shown to alter the differentiation of haematopoietic stem cells, promoting myelopoiesis and enhancing the activation status of bone marrow-derived macrophages [12]. In addition, compared to subcutaneous immunization, BCG delivered intravenously could be detected in the bone marrow for 7 months after BCG vaccination, suggesting prolonged interaction with the immune system. However, giving BCG intravenously in man is not likely to be practical and could induce adverse events including disseminated disease in immunosuppressed individuals.

Alternative delivery routes delivering BCG directly into the mucosa or lungs may also be worth exploring [55]. Compared to intravenous immunization, aerosol vaccination with BCG gave less bacterial dissemination and reduced bacterial counts in the lungs [56]. In mice, intranasal BCG induced better protection in the lungs than the standard intradermal vaccination, with induction of antigen-specific tissue-resident T-cells expressing a PD-1^+^ KLRG1^-^ cell-surface phenotype [57]. BCG can also induce protection in mice when given by the sublingual route [58]. In NHPs, mucosal delivery was associated with improved Th17 cell and IL-10 responses, slower IGRA conversion, lower pathology in the lungs and better control of *M. tuberculosis* growth in the lungs or lymphoid tissues compared to the intradermal route [59]. We still need a better understanding of how to maximise beneficial immune responses in the lungs while avoiding excessive immune activation.

## 9 Do different routes of administration also affect induction of innate training?

BCG vaccination can also induce non-specific protection against unrelated pathogens [60], [61] and reduce all-cause mortality in infants [62], [63], [64], [65], [66]. Importantly, this vaccine can induce a phenomenon known as “trained immunity”, resulting in epigenetic or metabolic reprogramming of the innate immune cells and enhanced surface marker expression or cytokine responses upon secondary stimulation [33], [67], [68], suggesting that this mechanism can contribute to the non-specific effects of BCG and protection against infectious diseases [69]. Adults vaccinated with BCG and then given yellow fever vaccine were shown to have reduced viraemia compared to BCG-naïve controls and this effect was associated with enhanced IL-1β production [70]. Infant BCG vaccination studies in Guinea-Bissau and the UK, as well as Australia demonstrated that production of cytokines associated with innate immunity was enhanced in BCG vaccinated infants compared to unvaccinated controls upon secondary stimulation with heterologous stimuli [32], [71], [72], [73]. This phenomenon is not restricted to BCG alone as another live mycobacterial vaccine – MTBVAC has been shown to enhance innate cytokine responses to LPS in human monocytes and in mice to improve resistance to an otherwise lethal infection with *S. pneumoniae* [74].

BCG revaccination was able to increase reversion of interferon-gamma release assay (IGRA) positivity in South African adolescents who had been BCG vaccinated at birth [75], which has led to renewed interest in giving a repeat BCG vaccination. In children, two randomised trials have provided some evidence that a repeat BCG vaccination may reduce all-cause mortality [76]; for example, in infants in Guinea Bissau who had received their diptheria, pertussis and tetanus (DPT) booster before their BCG revaccination at 19 months, there was some evidence of a reduction in mortality between 19 and 60 months [77]. In the South African H4/BCG trial, it was observed that the BCG revaccinated group had a lower rate of upper respiratory tract infections than in either the H4:IC31 group or the placebo group [75]. In an Indonesian study in which BCG was given monthly for 3 months to elderly individuals, the prevalence of acute respiratory infections was reduced [78]. These studies indicate that revaccination or boosting as well as primary vaccination with BCG may be able to induce or enhance innate memory with beneficial effects on survival; similar effects have also been observed with other live attenuated vaccines such as smallpox or oral polio vaccine [76].

The route of BCG administration can affect mycobacteria-specific immune responses and efficacy of the BCG vaccine. However, can different routes of BCG delivery affect the extent of innate immune training? So far, most studies of BCG-dependent innate immune training in humans have used intradermal BCG vaccination. However, recent exposure to tuberculosis has also been associated with innate immune training, suggesting that aerosol or mucosal interaction with mycobacteria can imprint innate immune responses [34]. Immunising calves with aerosolised BCG was associated with induction of trained immunity in PBMCs, although cytokine production by alveolar macrophages was not affected [79]. In humans, alveolar macrophages expressed lower levels of activation markers CD11b and HLA-DR after intradermal immunisation with BCG, although this study did not examine BCG-dependent changes in alveolar macrophage cytokine responses [80]. It is possible that induction of trained immunity in the lungs might be regulated or contributed to by adaptive immune cells, as adenovirus-dependent priming of alveolar macrophages in mice was found to be dependent on IFNγ produced by CD8+ T-cells in a model of *S. pneumoniae* infection [81].

Is this different if the BCG vaccine is delivered by other routes? In mice, intravenous delivery of BCG induced stronger haematopoietic cell expansion and differentiation compared to subcutaneously injected vaccine and was capable of priming bone marrow derived macrophages (BMDMs), enhancing their ability to control *M. tuberculosis* growth *in vitro* [12]. Intradermal BCG vaccination of humans also polarised haematopoietic stem cell differentiation into myeloid cells [82], suggesting that some BCG associated changes in the innate immune system can occur irrespective of the delivery route.

In another study, mice were vaccinated with BCG subcutaneously and their ability to control growth of *M. tuberculosis* in the lungs was compared with other routes of immunisation: intravenous, intranasal, aerosol or intramuscular [83]. A protective effect of similar extent on mycobacterial growth in the lungs was found for most immunisation routes despite varying colony forming units of BCG, except for low-dose aerosolised BCG which did not induce protection. In subcutaneously vaccinated mice, the protection against *M. tuberculosis* growth in the lung was independent of T-cell responses, suggesting that BCG mediated protection via innate immune cells [83]. Interestingly, depletion of neutrophils in this model was associated with diminished protection by BCG [83], supporting findings in humans, where intradermal BCG was associated with a neutrophil transcriptional signature and elevated neutrophil counts in BCG-vaccinated infants [82].

There also seem to be differences in how BCG, delivered via the skin, affects the innate immune cells. In humans, intradermal BCG vaccination induced a trained phenotype in monocytes in NOD2 dependent manner, enhancing accessibility of proinflammatory genes for transcription and cytokine production upon secondary stimulation with mycobacterial or heterologous antigens [67], [82], with similar changes happening in the NK cells [33] and NK cell cytokine responses associated with inhibition of mycobacterial growth years in these historically vaccinated individuals [31]. However, control of *M. tuberculosis* growth in lungs of subcutaneously BCG-vaccinated mice was not mediated by NOD2 dependent pathways, monocytes or NK cells [83]. Further investigation would be required to clarify whether these differences reflect the influence of route of vaccine delivery or differences in human and murine trained innate responses, as differences in regulation of trained immunity by long non-coding RNAs in human and murine models have been reported previously [84].

## 10 How can what we have learnt about BCG accelerate the development of new TB vaccines?

There is a pipeline of candidate TB vaccines in development, of varying types. Some are recombinant BCG vaccines, designed to be safer in infants who are HIV infected, or to induce improved protection by inclusion of additional antigens from *M. tuberculosis*. Some are other live mycobacterial vaccines, including *M. tuberculosis* itself with mutations that reduce its virulence, or environmental non-tuberculous mycobacteria. It is likely that any issues that affect growth of BCG bacilli in a BCG vaccine, will similarly affect the growth of another live mycobacterial vaccine. Other vaccine candidates include subunit or recombinant proteins in adjuvant, which would be given as a booster vaccine following BCG vaccination, that would depend on BCG vaccination having induced an effective primary immune response. Similarly, the vaccines that consist of viral vectors that deliver one or more antigens, are usually intended to boost a pre-existing immune response rather than induce a primary immune response. The TB vaccine portfolio is therefore very dependent on what BCG vaccination does or does not do. It may also be beneficial if primary vaccination (for example with a live mycobacterial vaccine) can induce non-specific innate training.

One surprising result from a recent vaccine trial of the subunit H4 vaccine to prevent infection rather than disease, was that repeat BCG given as the control arm, was more effective at inducing reversion to IGRA-negative status than the subunit vaccine (although neither vaccine provided significant protection against IGRA conversion, taken as indication of *M. tuberculosis* infection) [75]. This was surprising because in a number of earlier studies performed in different settings such as Malawi [23] or Brazil [85], there was no improvement seen with a repeat BCG vaccination although with data from a longer-term follow-up of the BCG-REVAC trial there was some evidence that repeat BCG could be protective in an area of Brazil with low prevalence of non-tuberculous mycobacteria [86].

## 11 Could inducing greater innate training improve the protection given by BCG or these new vaccines?

Trained immunity has been associated with protection against heterologous infections and has been implicated in the non-specific effects of BCG. However, improving innate immunity may also be able to enhance the protective efficacy of new TB vaccines [87], [88]. BCG could be detected in the bone marrow 7 months after BCG vaccination and where it could reprogram haematopoietic stem cells (HPSCs) inducing their differentiation into epigenetically primed myeloid cells capable of reducing growth of *M. tuberculosis* [12]. In another mouse immunisation model, BCG vaccination induced protection against *M. tuberculosis* in a neutrophil-dependent manner [83]. Of interest, BCG vaccination of human adults induced transcriptional signatures consistent with myeloid cell differentiation or neutrophil responses, epigenetically imprinting both the HPSCs and CD14+ cells [82]. Not only BCG, but the recombinant *M. tuberculosis* vaccine, MTBVAC, has also been shown to induce trained immunity *in vitro*, resulting in elevated production of IL-1β, TNFα or IL-6 upon secondary stimulation [74]. BCG-dependent enhancement of these cytokines could be exploited, as the cytokines could act as adjuvants to induce improved Th1 or Th17 responses that are considered protective against TB. Mycobacterial component-based vaccines, such as RUTI have also been shown to improve inhibition of mycobacterial growth *ex vivo* in association with phenotypic changes in monocytes from vaccinated mice [89]. Metabolites, such as fumarate, or the fungal component β-glucan, can also induce innate immune training *in vivo* [90], [91], [92]. BCG and β-glucan can induce features of trained immunity in cells from both neonates and adults [93], suggesting that microbial components, and metabolites might be exploited in combination with BCG or other anti-TB vaccines to enhance innate immunity and possibly protective T-cell responses not only in adults, but also in neonates, the main target group for immunisation against TB.

It may also be necessary to optimise vaccine regimens to maximise innate training. Just as for adaptive T-cell (and antibody) responses, BCG-dependent innate training (or that induced by other live vaccines such as MTBVAC [74] may be susceptible to external factors, resulting in variability. In healthy adults, circadian rhythms have been shown to modulate both heterologous and mycobacteria-specific cytokine production, with individuals administered BCG vaccine in the morning showing higher differences from baseline at 2 weeks or 3 months post-immunisation than individuals vaccinated in the evening [94]. What is learnt from BCG may help in the design of better vaccination strategies for both tuberculosis and other diseases [95].

It is also possible that trained innate or heterologous effects of BCG might be sex-specific, enhancing some immune responses more in males than in females or vice versa [96], [97]. It should be noted that such effects are subtle and often result in trends rather than large-scale effects on all-cause morbidity or mortality [98]. While neither the systematic review by the WHO SAGE committee in 2014, nor its update in 2016 found sex-differential effects on all-cause mortality in BCG-vaccinated infants [63], [64], some recent studies showed that beneficial effects of BCG on all-cause mortality can be observed at different time points since vaccination in males and females [65].

Finally, it is not fully clear for how long the effects of trained immunity last. Some studies in healthy adults showed that enhancement of cytokine production is transient and unlikely to last beyond a few months after vaccination [11], although as noted above some longer term protection may be induced [13]. However, while variability and lack of longevity of trained immunity might limit prime-boost strategies, vaccines, compounds and metabolites inducing this phenomenon could still be exploited as adjuvants. This rationale underpins new trials of BCG vaccination as an interim protective measure against COVID-19, for example in front-line healthcare workers [99], [100].

## 12 Conclusions

The BCG vaccine has been used for almost one hundred years but we still have a lot to understand about it [101]. BCG vaccination can induce long-lasting protection against tuberculosis, and induces T-cell responses, but there is considerable variability in individual responses to vaccination between and within different settings, which may result in both BCG itself and other factors affecting responses to vaccination [102]. Measuring growth inhibition of BCG or *M. tuberculosis* itself may be more informative but we still lack proven correlates of protection. New routes of administration are being investigated such as giving BCG intravenously or by aerosol. BCG revaccination is also attracting interest. We need to understand better what BCG does and does not do, in order to develop more effective vaccination regimes to protect against tuberculosis, using either BCG, a modified BCG vaccine, or a new TB vaccine. We also need to investigate whether increasing innate training might enhance the efficacy of BCG vaccination. Finally, when developing new vaccines, we need to avoid the loss of any beneficial non-specific protective effects that BCG vaccination provides to infants.

## Declaration of Competing Interest

The authors declare that they have no known competing financial interests or personal relationships that could have appeared to influence the work reported in this paper.
