# Supplementary material for: Can what have we learnt about BCG vaccination in the last 20 years help us to design a better tuberculosis vaccine?
Source: Vaccine. 2022 Mar 8;40(11):1525–33. doi: 10.1016/j.vaccine.2021.01.068 (PMC8899334; doi:10.1016/j.vaccine.2021.01.068)
Supplement: Supplementary data 1 [file mmc1.docx]

Dockrell Data statement

This review contains no original data.
